# Supplementary material for: Comparison of measured LDL cholesterol with calculated LDL-cholesterol using the Friedewald and Martin-Hopkins formulae in diabetic adults at Charlotte Maxeke Johannesburg Academic Hospital/NHLS Laboratory
Source: PLoS One. 2022 Dec 14;17(12):e0277981. doi: 10.1371/journal.pone.0277981 (PMC9749991; doi:10.1371/journal.pone.0277981)
Supplement: S1 Fig — (A-B) LDL-C of <1.4 mmol/L. (C-D) LDL-C of 1.4–1.7 mmol/L. (E-F) LDL-C of 1.8–2.5 mmol/L. (G-H) LDL-C of 2.6–2.9 mmol/L and (J-K) LDL-C ≥3.0 mmol/L. The plots show the regression line (Solid blue line) and the confidence interval for the regression line (dashed lines). (DOCX) [file pone.0277981.s001.docx]

(A)


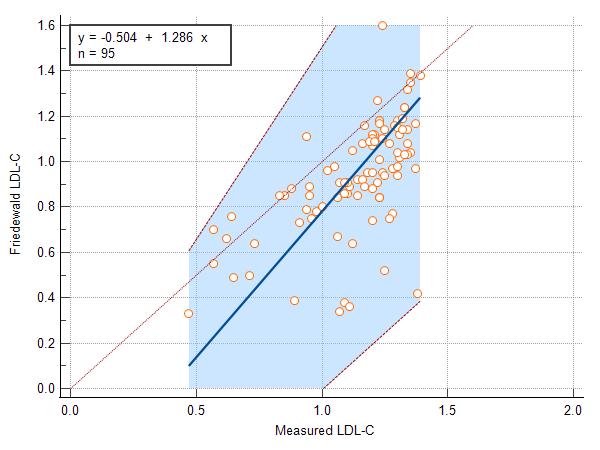


(B)


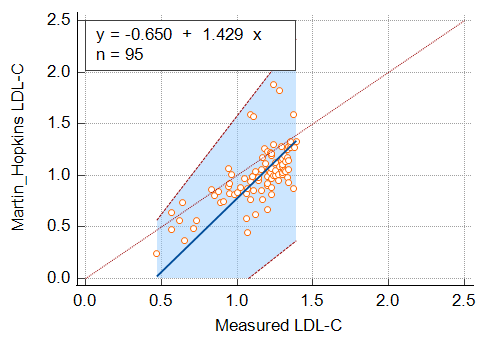


(C)


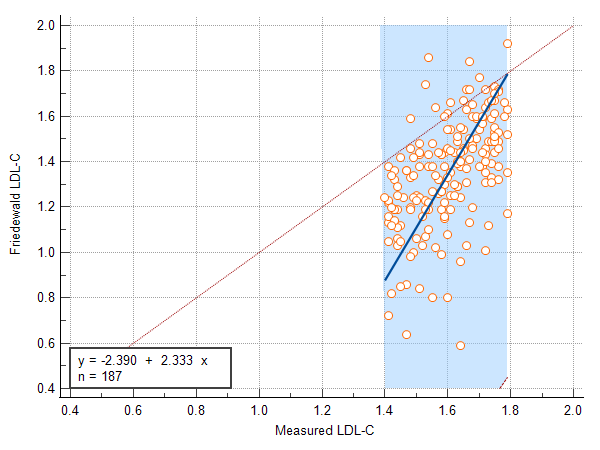


(D)


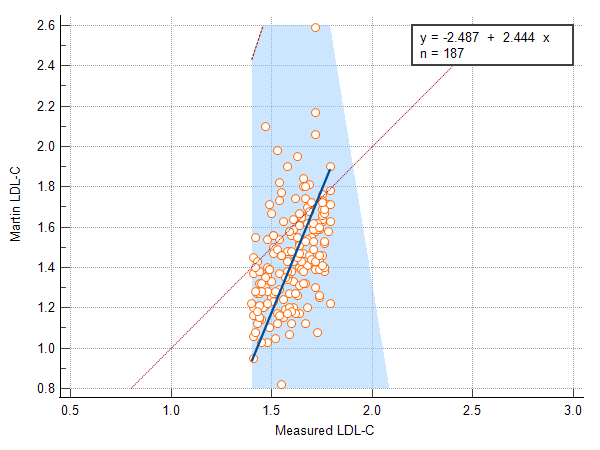


(E)


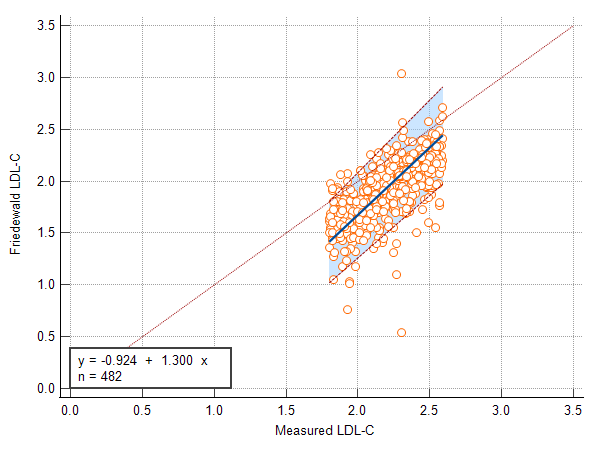


(F)


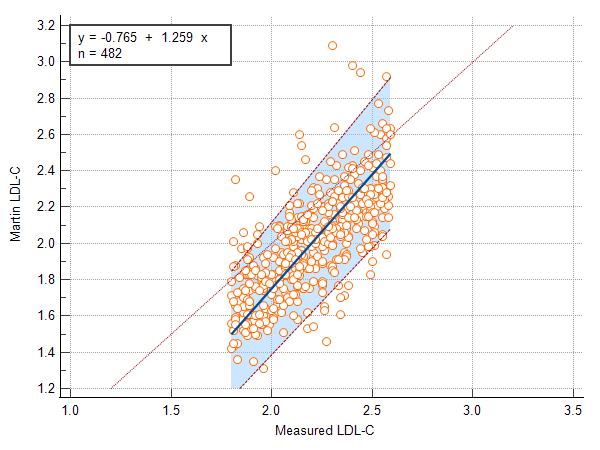


(G)


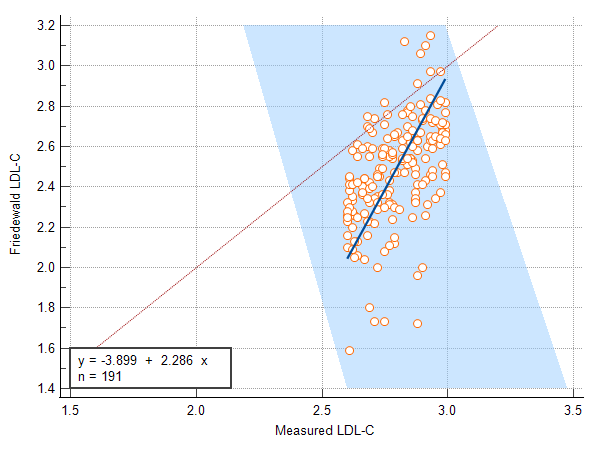


(H)


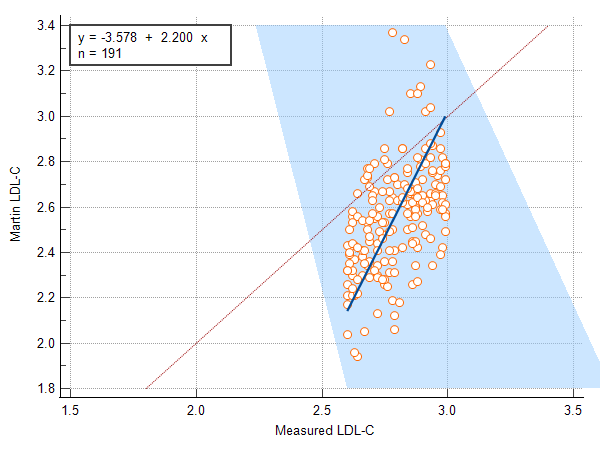


(I)


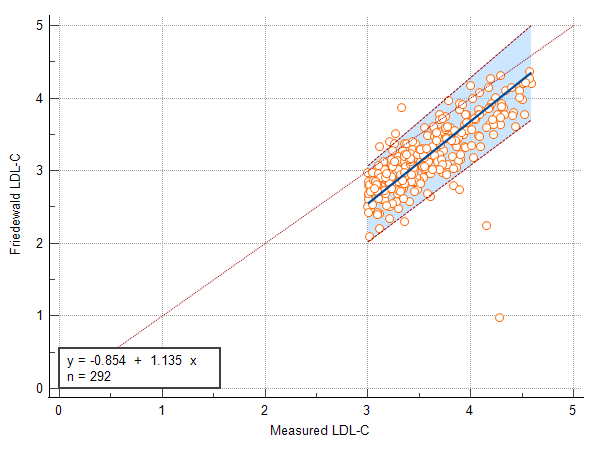


(J)


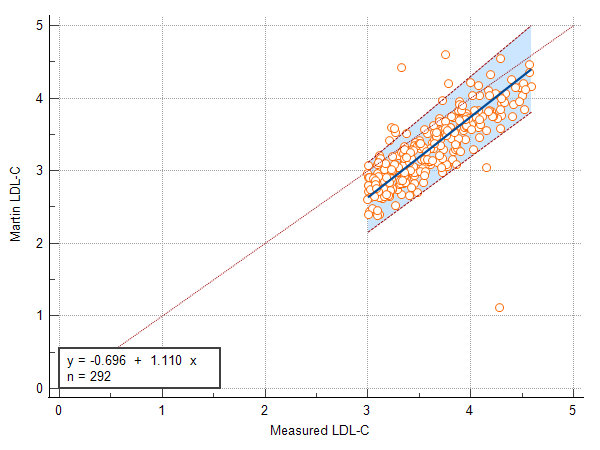


**Supplementary Fig: Passing-Bablok plots at different low-density lipoprotein (LDL-C) treatment target concentrations using the Friedewald as well as the Martin-Hopkins formulae**. (A-B) LDL-C of <1.4 mmol/L. (C-D) LDL-C of 1.4-1.7 mmol/L. (E-F) LDL-C of 1.8-2.5 mmol/L. (G-H) LDL-C of 2.6-2.9 mmol/L and (I-J) LDL-C ≥3.0 mmol/L. The plots show the regression line (Solid blue line) and the confidence interval for the regression line (dashed lines).
