# Supplementary material for: Comparison of measured LDL cholesterol with calculated LDL-cholesterol using the Friedewald and Martin-Hopkins formulae in diabetic adults at Charlotte Maxeke Johannesburg Academic Hospital/NHLS Laboratory
Source: PLoS One. 2022 Dec 14;17(12):e0277981. doi: 10.1371/journal.pone.0277981 (PMC9749991; doi:10.1371/journal.pone.0277981)
Supplement: Attachment — Submitted filename: Response to Reviewers.docx [file pone.0277981.s003.docx]

**A REBUTTAL LETTER**

Dear Shukri AlSaif

We thank you for giving us the opportunity to submit a revised version of the manuscript titled Comparison of measured LDL cholesterol with calculated LDL-cholesterol using the Friedewald and Martin-Hopkins formulae in diabetic adults at Charlotte Maxeke Johannesburg Academic Hospital/NHLS Laboratory to *PLOS ONE.*

We are grateful for the time and effort taken by you as well as the reviewer to give valuable feedback on the above titled manuscript.

Below is point-form response to the editor’s and reviewer’s comments.

- **Editor Point P 0.1**- Please ensure that your manuscript meets PLOS ONE's style requirements, including those for file naming.

**Response:** Thank you for providing us with the relevant templates for the requirements of PLOS ONE for research report. We have changed all major headings to font size 18 and also wrote them into sentence case. Figure captions changed as per PLOS ONE requirements, please see Results section page 9 to 14. Equations were formatted using the equation tool, see page 8. Table titles changed to bold. In-site citation changed to square brackets.

- **Editor Point 0.2**- Thank you for stating the following in your Competing Interests section:  
  "The authors state no conflict of interest."

  Please complete your Competing Interests on the online submission form to state any Competing Interests. If you have no competing interests, please state "The authors have declared that no competing interests exist.", as detailed online in our guide for authors at http://journals.plos.org/plosone/s/submit-now 
  This information should be included in your cover letter; we will change the online submission form on your behalf

**Response:** Thank you for the above suggestion. We have included the “The authors declare that no competing interest exist” in the cover letter. We appreciate that you will change this on the online submission form on our behalf.

- **Editor Point P0**.3- We note that you have stated that you will provide repository information for your data at acceptance. Should your manuscript be accepted for publication, we will hold it until you provide the relevant accession numbers or DOIs necessary to access your data. If you wish to make changes to your Data Availability statement, please describe these changes in your cover letter and we will update your Data Availability statement to reflect the information you provide.

**Response:** We confirm that data mentioned in the manuscript will be made available and relevant accession numbers will be provided.

- **Editor Point P 0**.4- Please review your reference list to ensure that it is complete and correct. If you have cited papers that have been retracted, please include the rationale for doing so in the manuscript text, or remove these references and replace them with relevant current references. Any changes to the reference list should be mentioned in the rebuttal letter that accompanies your revised manuscript. If you need to cite a retracted article, indicate the article’s retracted status in the References list and also include a citation and full reference for the retraction notice.

**Response:** Thank you. No papers in the references have been retracted.

Point-By-Point Reply to Reviewer’s Comments

- **Reviewer Point P 1.1-** Have the authors made all data underlying the findings in their manuscript fully available?

Reviewer #1: No

**Response:** Thank you very much for pointing it out. All raw data is uploaded on protocols.io

DOI: dx.doi.org/10.17504/protocols.io.yxmvm2ky6g3p/v1

(Private link for reviewers: [https://www.protocols.io/private/37E720DD488211EDB54B0A58A9FEAC02](https://www.protocols.io/private/37E720DD488211EDB54B0A58A9FEAC02%20) to be removed before publication.)

- **Reviewer Point P 1.2-** Review Comments to the Author

Reviewer #1: This is a well written manuscript which seek to compare measured LDL-C with calculated LDL-C using the Friedewald and
Martin-Hopkins formulae in diabetic patients over a range of LDL-C and TG concentrations. The limitations of the Friedewald equation is well documented, subsequently, many studies has evaluated other formulae and compared with the Friedewald equation.
the current manuscript is well written, I however have the following questions or remarks;
did authors consider the medication of study participants?
did authors consider the disease duration? these factors are confounding and authors should answer and address their consequence on their lipid profile
Figure legends/titles should be below the figures and not above it, authors should revise as such.

**Response:** Thank you for the positive feedback and highlighting the questions above. This was a retrospective study with limited access to participants’ medical records thus drug history and disease duration could not be obtained, therefore this was not assessed. We aimed to compare the analytical performance of measured LDL-C to calculated LDL-C over a wide range of LDL-C and triglyceride concentrations. We do acknowledge the effect of lipid lowering drugs on the lipid profile however they do not impact the analytical performance of LDL-C methods.

We have included our limited access to medical records as a limitation to our study and in addition recommended that further studies include participants’ drug history.

Figure titles and legends placement have been changed as suggested.
